# INCIDENCE OF SMALL FOR GESTATIONAL AGE NEONATES, ACCORDING TO THE FENTON AND INTERGROWTH-21^ST^ CURVES IN A LEVEL II MATERNITY

**DOI:** 10.1590/1984-0462/2021/39/2019245

**Published:** 2020-07-03

**Authors:** Claudia Malisano Barreto, Marley Aparecida Lambert Pereira, Anna Carolina Boni Rolim, Samira Ali Abbas, Dante Mario Langhi, Amélia Miyashiro Nunes dos Santos

**Affiliations:** a Universidade Federal de São Paulo, São Paulo, SP, Brazil.; b Irmandade da Santa Casa de Misericórdia de São Paulo, Hospital São Luiz Gonzaga, São Paulo, SP, Brazil.; c School of Medical Sciences, Santa Casa de São Paulo, São Paulo, SP, Brazil.

**Keywords:** Infant, newborn, Infant, small for gestational age, Fetal growth retardation, Incidence, Recém-nascido, Recém-nascido pequeno para a idade gestacional, Restrição de crescimento intrauterino, Incidência

## Abstract

**Objective:**

To compare the incidence of small for gestational age infants among late preterm and term newborns, using the Fenton and Intergrowth-21^st^ curves.

**Methods:**

Observational and retrospective study with newborns in a level II maternity. The study was approved by the Institution’s Ethics Committee. Live births from July 2007 to February 2009 with a gestational age from 34 to 41 weeks and seven days were included. Neonates with incomplete data were excluded. Appropriate weight for gestational age was assessed by the Fenton and Intergrowth-21^st^ intrauterine growth curves, considering birth weight <10^th^ percentile as small for gestational age. The degree of agreement between the two curves was assessed by the *Kappa* coefficient. Numerical variables were compared using the Student t-test or the Mann-Whitney. Categorical variables were compared using the chi-square test. Statistical analyzes were performed using SPSS17^®^ software, considering significant, p<0.05.

**Results:**

We included 2849 newborns with a birthweight of 3210±483 g, gestational age of 38.8±1.4 weeks; 51.1% male. The incidence of small for gestational age in the full sample was 13.0 vs. 8.7% (p<0.001, *Kappa*=0.667) by the Fenton and Intergrowth-21^st^ curves, respectively. Among late preterm, the incidence of small neonates was 11.3 vs. 10.9% (p<0.001; *Kappa*=0.793) and among full-term infants it was 13.1% vs. 8.5% (p<0.001; *Kappa*=0.656), respectively for the Fenton and Intergrowth-21^st^ curves.

**Conclusions:**

The incidence of small for gestational age newborns was significantly higher using the Fenton curve, with greater agreement between the Fenton and Intergrowth-21^st^ curves among late preterm, compared to full term neonates.

## RESUMO

**Objetivo:**

Comparar a incidência de neonatos pequenos para idade gestacional entre nascidos vivos pré-termo tardios e a termo utilizando as curvas de Fenton e Intergrowth-21^st^.

**Métodos:**

Estudo observacional retrospectivo com recém-nascidos de uma maternidade pública de nível secundário. Foram incluídos nascidos vivos de julho/2007 a fevereiro/2009 com idade gestacional de 34 a 41 semanas e seis dias. O estudo foi aprovado pelo Comitê de Ética da instituição. Foram excluídos recém-nascidos com dados incompletos. Para adequação do peso/da idade gestacional, utilizaram-se as curvas de crescimento intrauterino de Fenton e Intergrowth-21^st^, considerando-se pequeno aquele com peso ao nascer <10º percentil. O grau de concordância entre as duas curvas foi avaliado pelo coeficiente Kappa. As variáveis numéricas foram comparadas pelo teste *t* de Student ou de Mann-Whitney, conforme distribuição, e as categóricas pelo teste χ^2^. As análises estatísticas foram realizadas no programa Statistical Package for the Social Sciences (SPSS) 17^®^, considerando-se significante p<0,05.

**Resultados:**

Foram incluídos 2.849 recém-nascidos com peso ao nascer de 3210±483 g, idade gestacional de 38,8±1,4 semanas, sendo 51,1% masculinos. A incidência de recém-nascidos pequenos para a idade gestacional pela curva de Fenton e Intergrowth-21^st^ na amostra total foi, respectivamente, de 13 e 8,7% (p<0,001; Kappa=0,667). Entre os pré-termo tardios, a incidência foi de 11,3 e 10,9% (p<0,001; Kappa=0,793) e entre os nascidos a termo foi de 13,1 e 8,5%, (p<0,001; Kappa=0,656), respectivamente, para as curvas de Fenton e Intergrowth-21^st^.

**Conclusões:**

A incidência de recém-nascidos pequenos para idade gestacional foi significantemente maior pela curva de Fenton, com maior concordância entre as curvas de Fenton e Intergrowth-21^st^ em recém-nascidos pré-termo tardios do que nos nascidos a termo.

## INTRODUCTION

Estimates show that 15 million premature babies^1^ and 32 million newborns (NBs) that are small for gestational age (SGA) are born annually in the world, corresponding to an incidence of 27% of SGA NBs among live births in low and middle income countries.^2^ In the Brazilian context, three birth cohorts from 1982, 1993 and 2004 from Pelotas, RS, showed an incidence of SGA infants, defined as birth weight below the 10th percentile of the Williams et al. Curve,^3^ of 14.8, 9.4 and 12%, respectively.^4^

 SGA NBs are five times more likely to die in the neonatal period and 4.7 times more likely to die in the first years of life when compared to newborns appropriate for gestational age.^5^ In addition, survivors may be compromised with regard to their growth, and the development of chronic diseases in adulthood, such as type 2 diabetes, systemic arterial hypertension, obesity and cardiovascular diseases.^6^ ^,^ ^7^ ^,^ ^8^

The growth curves differ from each other in accordance with the type of population included and the design of the study adopted. The reference curves, such as Fenton’s,^9^ describe the growth of a sample of children without characterizing it as a normal pattern, and using cross-sections. Another type of curve represents a supposedly normal growth pattern for a population, such as the Intergrowth-21^st^ curve.^10^ The Fenton curve^9^ is based on the analysis of various intrauterine growth reference curves treated by means of a meta-analysis, with the inclusion of patients from developed countries. The population pattern includes European, North American, Canadian and Australian mothers. The Intergrowth-21^st^ curve^10^ was constructed based on a prospective, multiethnic population study, including several countries, such as Brazil, Italy, Oman, the United Kingdom, the United States, China, India and Kenya, and it was designed specifically to create an international growth pattern.

The choice of a certain intrauterine growth curve, among the countless existing ones,^9^ ^,^ ^10^ ^,^ ^11^ ^,^ ^12^ ^,^ ^13^ can influence the incidence of SGA NBs in a population. Thus, in a retrospective cohort in Turkey, Tuzun et al.^14^ found, among 248 preterm newborns with a mean gestational age of 29.1±2.1 weeks, there was a significantly higher rate of SGA newborns using the Intergrowth-21^st^ curve^10^ compared to Fenton’s curve^9^ (respectively, 12 vs. 15%; p = 0.004).

Considering this issue, the objective of the present study was to compare the incidence of SGA NB between late and full-term preterm live births, in a secondary level maternity hospital, using Fenton’s intrauterine growth curve^9^ and the Intergrowth-21^st^curve.^10^

## METHOD

This is a retrospective observational study, with secondary analysis of data on live births from a public secondary maternity hospital, prospectively collected from July 2007 to February 2009, in the city of São Paulo, SP, Brazil. This maternity hospital is referred to for high-risk prenatal care and has 30 beds for joint accommodation. The neonatal unit has eight intermediate care beds and six intensive care beds.

This study was developed based on the project called *Evaluation of the frequency of neonatal alloimmune neutropenia in Brazilian newborns*, and received the NBs’ legal guardians’ signatures on the free and informed consent forms (FICF) (Research Ethics Committee - *Comitê de Ética em Pesquisa* - CEP - No. 0051/07). The current project was approved by the CEP of the Universidade Federal de São Paulo # 1,624/08, considering the FICF of the original project.

The inclusion criteria were: consecutive live births during the study period with a gestational age of 34 to 41 weeks and six days, and NBs whose parents or guardians signed the FICF for the original project. The chosen gestational age range aimed to include the common scope of the two curves proposed for the study. The exclusion criteria were: NB with missing gestational age, birth weight or sex. It is worth mentioning that, because the objective of the study was to verify the SGA incidence among live births, all live births were included.

The adequacy of weight to gestational age was assessed using the Fenton^9^ and Intergrowth-21^st^ curves,^10^ considering newborns appropriate for gestational age (AGA) those with a birth weight between the percentile 10 and 90. SGA was considered when birth weight was below the 10th percentile, and large for gestational age (LGA) was considered when weight was above the 90th percentile of those curves.^11^

Data on the maternal and newborn demographic and clinical characteristics of the original project’s Excel spreadsheets were analyzed. Such data had been collected according to the routine established in the hospital. Thus, maternal history was obtained from the parturient’s medical record, and was associated with maternal anamnesis and the consultation with the obstetrician on duty the day of the child’s birth. Demographic, clinical and anthropometric data were recorded by the pediatrician who received the newborn in the delivery room. Gestational age was assigned by the hospital’s neonatology team, which routinely considered the best obstetric estimate based on the date of the last menstrual period or ultrasound examination before 14 weeks.^10^ In the absence of such data, the pediatrician considered the gestational age as assessed by the New Ballard method.^15^

For the number of live births of 3,434 in the period of the study, a 95% confidence level, a maximum acceptable error of 1% and an estimated percentage of SGA newborns of 10 to 15% in the Brazilian population,^4^ ^,^ ^16^ ^,^ ^17^ ^,^ ^18^ the sample size calculated to estimate the incidence of SGA NBs was 1,723 to 2,019 live births.

The numerical variables were expressed as mean and standard deviation or median (minimum-maximum) and compared using the Student’s or Mann-Whitney test, according to the data distribution, assessed by the Kolmogorov-Smirnov test. Categorical variables were described in number and percentage and compared using the χ^2^ test. In order to verify the degree of agreement between the SGA incidences obtained by the Fenton and Intergrowth-21^st^ curves, the Kappa coefficient was calculated. According to the value of the Kappa coefficient, the following were considered: insignificant agreement (Kappa=0 to 0.20), median agreement (0.21 to 0.40), moderate (0.41 to 0.60), substantial (0.61 to 0.80) and almost perfect (0.81 to 1.00).^19^ Statistical analyzes were performed using the program Statistical Package for the Social Sciences (SPSS) 17^®^ (IBM SPSS Statistics, Somers, NY, United States). Statistical significance was set at p <0.05.

## RESULTS

**Figure 1:**
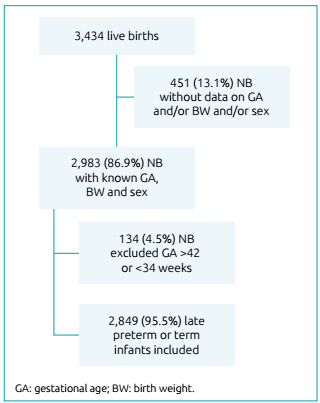
Flowchart of newborns included in the study.

During the study period, 3,434 live NBs were born, of which 2,983 (86.9%) had complete data on gestational age, birth weight and sex. Of these, 134 (4.5%) NBs were excluded because they had a gestational age of less than 34 weeks or equal to or greater than 42 weeks. Thus, 2,849 late or full-term preterm infants were included, corresponding to 95.5% of live births with a known gestational age, birth weight and sex (Figure 1).

The mean maternal age was 25.5±6.5 years (minimum=12.0; maximum=46.0). Regarding gestational history, the median number of pregnancies was 2.0 (range=1-16), and the median number of deliveries was 1.0 (range = 0-15). The number of abortions ranged from zero to four, the number of fetal deaths ranged from zero to three, and the number of live children ranged from zero to fifteen. A total of 2,663 (93.5%) pregnant women had prenatal care with an average of 5.8 ± 3.0 appointments (median = 6; range = 0-17).

With regard to morbidity, 3.8% of pregnant women had chronic arterial hypertension, 2.3% had pregnant hypertensive disease, 0.6% had diabetes mellitus, 1.2% had gestational diabetes, 1.4% had intrauterine growth restriction, 2.7% had a urinary tract infection, 0.5% had placenta previa and 0.3% had placental abruption. The type of delivery was normal in 1,858 (65.2%) pregnant women, forceps in 97 (3.4%), and cesarean sections in 894 (31.4%) pregnant women.

Of the 2,849 NBs assessed in the study, 1,455 (51.1%) were male, had a birth weight of 3210±483 g (minimum=1320 g; maximum=5270 g) and the first minute Apgar score was 8.1±1.4 and the 5th minute was 9.2±0.9. The mean gestational age was 38.8±1.4 weeks (minimum=34.0 weeks; maximum=41.9 weeks), with 221 (7.8%) late preterm infants and 2,628 (92.2%) born at term.

**Figure 2:**
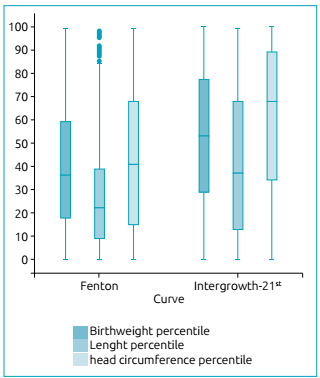
A boxplot with percentiles of birth weight, length and head circumference of newborns (n=2,849), showing significantly lower percentile values for the three parameters on the Fenton curve, compared to the Intergrowth-21^st^ curve (p <0.001).

The percentile of birth weight (39.8±25.6 *vs*. 52.3±28.4; p <0.001), length (26.9 ± 22.5 *vs*. 41.4 ± 30, 6; p <0.001) and head circumference (42.8 ± 29.5 *vs*. 58.7 ±, 2; p <0.001) of the total sample was, on average, lower in the newborns evaluated by the Fenton curve when compared to the results obtained by the Intergrowth-2^st^ curve (Figure 2).

There was a significant difference (p <0.001) and moderate agreement (Kappa=0.554; standard error=0.020) in the distribution of birth weight/gestational age of 2,849 NBs, according to the Fenton and Intergrowth-21^st^ curves. The distribution of 2,849 NBs using the Fenton curve was: AGA = 2,382 (83.6%; 95% confidence interval - 95% CI 82.2-84.9%), SGA=369 (13%; 95% CI 11.8-14.2%) and LGA=98 (3.4%; 95% CI 2.8-4.2%). Through the Intergrowth-21^st^ curve, 2,290 (80.4%; 95% CI 78.9-81.8%) were AGA, 247 (8.7%; 95% CI 7.7-9.8%) were SGA and 312 (11%; 95% CI 9.9-12.2%) were LGA.

**Figure 3:**
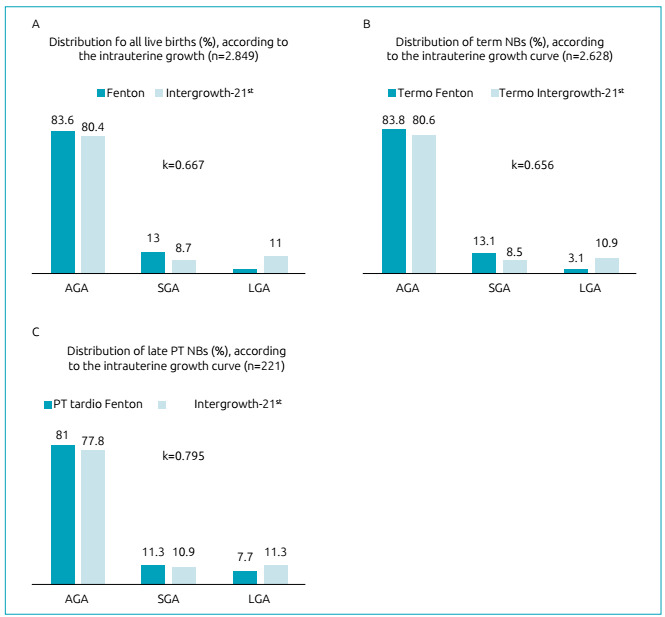
Distribution of percentages of live births, according to birth weight/gestational age, classified by the Fenton and Intergrowth-21^st^ curves in (A) total sample, (B) among NB births at term and (C) among preterm NB (PT). Kappa coefficients (k) refer to the degree of agreement only for the classification of small newborns for gestational age (SGA) between the two curves. AGA: NB appropriate for gestational age; SGA: Small NB for gestational age; LGA: Large NB for gestational age.

When only the incidence of SGA NBs was compared using the Fenton and Intergrowth-21^st^ curves (13 *vs*. 8.7%, respectively, p <0.001), there was agreement between the two curves in 216 (7.6 %; 95% CI 6.7-8.6%) cases and a Kappa coefficient=0.677 (standard error = 0.023) (Figure 3A).

The agreement between the two curves in the distribution of NBs was greater among late preterm NBs than between full-term NBs. Considering the 2,628 term newborns, according to the Fenton curve, the incidence of AGA newborns was 83.8% (95% CI 82.4-85.2%), for SGA it was 13.1% (95% CI 11.0 -14.4%) and for LGA it was 3.1% (95% CI 2.5-3.8%). Through the Intergrowth-21^st^curve, 80.6% AGA newborns (95% CI 79-82.1%), 8.5% SGA newborns (95% CI 7.5-9.6%) and 10.9 % LGA (95% CI 9.8-12.2%) (p <0.001; Kappa = 0.532, standard error = 0.021;) were detected. There was agreement between the two curves in relation to SGA newborns in 196 (7.5%) cases (Kappa=0.656, standard error = 0.024) (Figure 3B).

According to the Fenton curve, among the 221 late preterm infants, 81% were AGA (95% CI 75.3-85.6%), 11.3% were SGA (95% CI 7.8-16.0%) and 7.7% were LGA (95% CI 4.9-12.0). Through the Intergrowth-21^st^ curve, 77.8% were AGA (95%CI 71.9-82.8), 10.9% were SGA (95% CI 7.4-15.7) and 11.3% were LGA (95% CI 7.8-16.2) (p <0.001; Kappa=0.779; standard error=0.051). In 20 (9.0%) cases, there was agreement in the classification of SGA newborns using the two curves (Kappa=0.795, standard error=0.067) (Figure 3C).

The maternal and neonatal clinical characteristics of newborns classified as SGA by the two curves were similar, except for the greater proportion of males (57.2 vs. 48.6%; p=0.036) and higher birth weight values (2640±319 vs. 2464±312 g; p<0.001), length (46.4±21.3 vs. 45.6±2.3 cm; p<0.001) and head circumference (32.9±1.8 vs. 32.5± .7 cm; p=0.001) using the Fenton curve, as compared to the Intergrowth-21^st^ curve.

**Figure 4:**
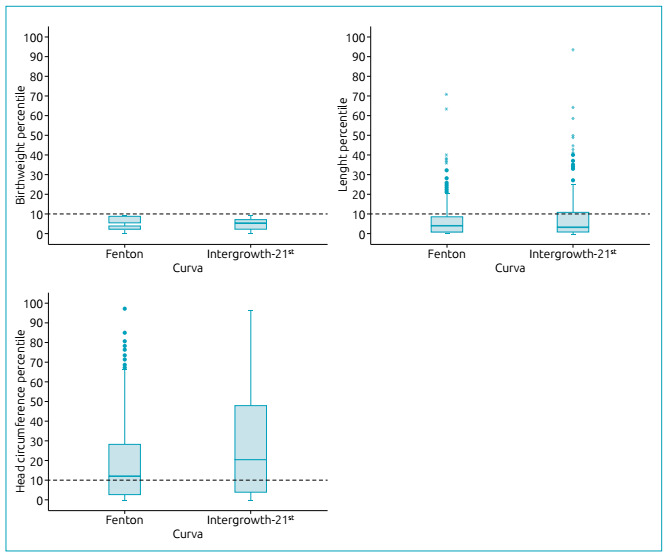
Boxplot of weight percentile (p = 0.796), length (p = 0.106) and head circumference (p <0.001) at birth among newborns that were small for gestational age, according to the Fenton (n=369) and Intergrowth-21^st^ curves (n = 247)

Among the SGA newborns, the mean and standard deviation of the birth weight percentiles (4.5±2.8 vs. 4.5±3.0; p=0.796) and length (7.1±9.3 vs. 8.4 ± 13.2; p = 0.106) were similar in the two curves, respectively, the Fenton vs. the Intergrowth-21^st^. However, it was observed that the percentile of head circumference (19.7±21.6 vs. 28.7±28; p<0.001) was lower when evaluated by the Fenton curve (Figure 4).

It was found that, among the SGA NBs, even when the birth weight was below the 10^th^ percentile, there was a very large variation in the percentile of length and head circumference, with approximately 25 and 50% of the SGA NB reaching percentiles of length and head circumference above the 10^th^ percentile (Figure 4).

The incidence of SGA/intrauterine growth restriction (IUGR) in the total number of newborns studied according to the adopted curve and the respective Kappa coefficients is shown in Table 1.

**Table 1:** Incidence of newborns (NB) that were small for gestational age (SGA) and/or suffered from intrauterine growth restriction (IUGR) and the degree of agreement between the Fenton and Intergrowth-21^st^ curves in the identification of SGA/IUGR newborns, according to the criterion used and the curve adopted (n = 2,849).

	Fenton	Intergrowth-21^st^	p**	Kappa***
BW <10^th^ percentile*	369 (13.0%)	247 (8.7%)	<0.001	0.554
BW <3^rd^ percentile*	112 (3.9%)	76 (2.7%)	<0.001	0.800
BW and length and HC <10^th^ percentile*	82 (2.9%)	71 (2.5%)	<0.001	0.445
BW<-2SD	81 (2.8%)	61 (2.1%)	<0.001	0.451

*Criteria from Beune et al.^29^: BW<10^th^ percentile=SGA NB; BW<3^rd^ percentile=IUGR; BW and length and HC<10^th^ percentile=IUGR; BW: birth weight; HC: head circumference; SD: standard deviation. **p=χ^2^ test; ***Kappa coefficient=0.41-0.60: moderate; 0.61-0.80: substantial; and 0.81-1.00: almost perfect.^19^

## DISCUSSION

The results of the present study showed incidences of SGA NBs between late and full-term preterm live births, in a secondary level maternity hospital, using Fenton’s intrauterine growth^9^ and the Intergrowth-21^st^ curves.^10^ There was a statistically significant difference in the incidences detected by the Fenton^9^ and Intergrowth-21^st^ curves,^10^ however the agreement between the two curves was substantial.

The incidence of SGA newborns found in this research is compatible with some other Brazilian studies.^4^ ^,^ ^16^ In Pelotas, Zambonato et al.^16^ found a 13.1% prevalence of SGA births, considering SGA to be birth weight below the 10^th^ percentile of the Williams curve.^3^ At Hospital das Clínicas of the School of Medicine of the Universidade de São Paulo, Rodrigues et al.^20^ observed an incidence of SGA newborns for the 38-41 week gestational ranges of 18.8 vs. 14.2%, and for 37-41 weeks gestational ranges of 24.3 and 15.2%, respectively, with the Alexander et al. ^13^ ^,^ ^12^ and Fenton curves.^9^ Such data showed an incidence close to that found in this study for the Fenton curve^9^ in term NBs.

However, other studies have found a higher incidence of SGA NB. For example, Teixeira et al.^17^ indicated a 17.9% frequency of SGA newborns in a public maternity hospital in São Paulo, using the curve of Alexander et al.^12^ Kozuki et al.^21^ reached a 23.7% prevalence of SGA newborns using the Intergrowth-21^st^ curve,^10^ compared with 32.8% using the Alexander et al. curve^12^ and 36% using the Oken et al. curve^22^ in 16 prospective cohorts from ten low- and middle-income countries. This shows that studies using the Alexander et al. curve^12^ had a higher incidence of SGA NBs, possibly because it is a personalized curve for the American population, with greater weight and height than most of the other populations evaluated. This demonstrates the influence of population type included in the construction of the curves with regard to the incidence of SGA NB. In New Zealand, Anderson et al.^23^ found a 4.5% SGA NB incidence through using the Intergrowth-21^st^ curve and 11.6% through using Gardosi et al.^24^ In a population study in Kobe, Japan, with 27,288 children, the prevalence of birth weight or length for gestational age below -2 standard deviations was 3%.^25^ Therefore, the incidence of SGA infants depends on the definition criteria, the selection of the curve used, and the socioeconomic, environmental and health conditions of the population studied.

In this sense, in the present study, there was a difference in incidence identified by the Fenton curve^9^ and by that of Intergrowth-21^st^,^10^ since the models for the construction of these curves differed.

In addition, another factor that may have contributed to the difference in the incidence of SGA NB between the two curves is the composition of the gestational age ranges included in its construction. The Intergrowth-21^st^ curve^10^ covered a small number of newborns with a gestational age of less than 33 weeks, and its accuracy in classifying the neonatal population was limited to gestational ages of less than 33 weeks. This restriction no longer exists today, because, subsequently, an extension of the curve for extreme premature infants was published using the same methodology as the original curve, and was available for use in premature infants with 24 weeks of gestational age or more.^26^

The Fenton curve^9^ mirrors the intrauterine curves between 24 and 36 weeks of gestational age, however, after 36 weeks, it shows important differentiation in growth, perhaps due to the physiological aspects of postpartum newborns. In addition, the mathematical process of smoothing the Fenton curve^9^ may not actually reflect normal growth after 36 weeks of gestational age, because it was constructed with cross-sectional data of children born prematurely and, as such, does not correspond to normal conditions.^27^ This limitation can be observed in the present study, when the incidence and degree of agreement between term and late preterm newborns were compared separately. In the present study, the difference in the incidence of SGA NBs between late preterm NBs decreased from 11.3% using the Fenton curve to 10.9% using the Intergrowth-21^st^ curve, and the degree of agreement increased (Kappa=0.793). However, among term NBs, the difference in the incidence of SGA NBs between the two curves persisted (13.1 vs. 8.5%), and the degree of agreement was lower than in preterm (Kappa=0.656). These results suggest that the two curves similarly assess gestational weight/age adequacy and show less agreement when it comes to term pregnancy.

Thus, despite the difficulty in choosing the best intrauterine growth curve, in our context the standard Intergrowth-21^st^ curve^10^ seems to be a better option for late and full-term preterm infants, since this curve was carefully standardized in order to reflect standard intrauterine growth. It included Brazilian pregnant women, and women from other nationalities, and it contemplated the gestational age range studied here. Thus, although the objective of the present study was only to compare the incidences of SGA NBs detected by the two curves, because of the population characteristics and the gestational age range evaluated, in addition to the specificities of the construction of the curves used, it can be assumed that the incidence of SGA NBs in the population studied would be 8 to 10%, according to the 95%CI of the incidence detected by the Intergrowth-21^st^ curve.^10^

Among the SGA newborns identified by the two curves, length was also below the 10th percentile in about 75% of cases. Head circumference was above the 10th percentile in more than 50% of cases, with some NBs reaching the 98th to 99th percentiles on the Fenton^9^ and Intergrowth-21^st^ curves,^10^ respectively, suggesting a brain protection mechanism. Such data refer to the need to differentiate the SGA NBs in isolation from the ones who suffered from IUGR, to define follow-up strategies.^25^ ^,^ ^28^ As such, the use of the definition criteria for IUGR by Beune et al.^29^ showed that, among the SGA newborns, 22 to 30% presented IUGR, making the incidence of 2.5 to 3.9% of newborns with IUGR depending on the criterion and curves adopted. Such data show that, although the sample of the present study was collected in a secondary-level hospital, the frequency of maternal morbidity and IUGR was relevant, possibly because it meets high-risk prenatal care. In addition, the incidence of SGA NBs among full-term NBs found here was close to that observed at the Hospital das Clínicas from the School of Medicine of the Universidade de São Paulo,^20^ when the Fenton curve was used ^9^

As limitations of the study, it is possible to consider the retrospective design and the analysis of secondary data, which may have compromised the accuracy of the collected data. Another limiting factor was the fact that the sample studied was collected ten years ago and in a single center, with no guarantees of external validity of the study. As positive points, we highlight the sample size and the inclusion of 95% of live NBs who met the inclusion criteria, in addition to the sample calculation, which ensured the internal validity of the study.

In conclusion, it can be said that the incidence of SGA newborns ranged from 8.7 to 13%, with a significant difference in incidence and moderate agreement in the distribution of birth weight adequacy to gestational age, assessed by the Fenton^9^ and Intergrowth-21^st^ curves.^10^ Furthermore, the agreement in the identification of SGA newborns is higher among late preterm newborns when compared to that observed in term newborns.
